# Experience With the Lattice Technique of Palliative Radiotherapy for Treating Voluminous Metastases of Renal Cancer in the Radiotherapy Department of Solca Quito, Ecuador: A Case Report

**DOI:** 10.7759/cureus.89473

**Published:** 2025-08-06

**Authors:** Raul Puente-Vallejo, María del Pilar Pacheco, Iqrah Muhammad

**Affiliations:** 1 Radiation Oncology Department, Hospital Metropolitano, Quito, ECU; 2 Palliative Care Department, Hospital Solca Nucleo de Quito, Quito, ECU; 3 College of Health Sciences, Universidad San Francisco de Quito, Quito, ECU

**Keywords:** bulky tumor, clear renal cell carcinoma, lattice radiotehrapy (lrt), metastasis, spatially fractionated radiotherapy (sfrt)

## Abstract

Lattice radiotherapy (LRT) is a type of spatially fractionated radiation therapy (SFRT) that enables the delivery of ablative doses to specific internal regions of large tumoral lesions, while surrounding tissues and nearby critical structures receive significantly lower exposure. This technique relies on a spatial distribution strategy that allows dose levels of radiation to be applied within the tumor in a single session or, alternatively, over the course of five sessions. Over time, LRT has gained attention as a promising method for managing large tumors, especially in cases where conventional treatments may pose higher risks or be less effective, offering the benefit of reduced side effects. This report details the case of a 67-year-old patient suffering from metastatic kidney cancer with a large retroperitoneal mass, which led to severe abdominal pain and symptoms from compression of the inferior vena cava. The patient was treated with LRT over five sessions. The intervention led to rapid relief of symptoms and, during follow-up, a noticeable reduction in tumor size was observed, contributing to a clear improvement in the patient’s daily functioning and quality of life. This clinical experience emphasizes the potential role of LRT in addressing large, hard-to-treat tumors and suggests that it may be an effective palliative option in advanced cases of kidney cancer.

## Introduction

Renal cell carcinoma (RCC) is the most common renal cancer, accounting for 80-85% of primary renal neoplasms, and is responsible for more than 10,000 deaths per year, being the most lethal urological malignancy [1]. Its most frequent subtype is clear cell (75-80%); while it occurs in a localized form in most cases, it manifests as advanced or metastatic disease in 20-30% of cases [2]. Treatment of metastatic disease is primarily palliative: the goal is to relieve symptoms and improve the patient's quality of life rather than to eliminate the disease [3]. Immunotherapy has emerged as a promising treatment. Immune checkpoint inhibitors (ICIs) have demonstrated clinical efficacy as monotherapies and in combination regimens with antiangiogenic agents. Other immunomodulators, radiotherapy techniques, and other treatment modalities are currently areas of active investigation [4]. Bulky tumors and those considered radioresistant, such as adenocarcinomas, sarcomas, gliomas, and melanomas, pose a therapeutic challenge [5], especially in radiation oncology, when surgical resection is not possible [6]. Delivering a homogeneous dose in these tumors presents various challenges, such as tumor heterogeneity and beam modulation, due to the size and volume of the tumor, and the area of irradiation [7].

To overcome these limitations, spatially fractionated radiation therapy (SFRT) has emerged as a promising strategy for large tumors [8]. SFRT uses alternating high- and low-dose regions within the target volume to safely escalate dose while minimizing toxicity to surrounding healthy tissue [8]. The resulting dose pattern is deliberately heterogeneous and geometrically organized, similar to a carefully arranged orchard in which robust, tall trees (representing high-dose regions) are planted at regular intervals across a field of low groundcover (representing the low-dose background). When planned using a two-dimensional (2D) technique, SFRT is known as GRID radiotherapy. This method, employing either precast blocks or multileaf collimators (MLC), creates a pattern of high-dose "peaks" and low-dose "valleys" across the tumor, and can provide timely symptom management in the palliative setting [8,9]. However, 2D GRID may expose significant volumes of normal tissue to radiation and often delivers the highest dose to superficial areas outside the clinical target [10].

While GRID is more widely available, recent advancements in SFRT techniques have led to the transition from 2D GRID to a 3D configuration, known as Lattice [8]. Lattice radiotherapy (LRT) generates multiple high-dose vertices (small spherical regions) embedded in a lower dose background that covers the entire gross tumor volume [8]. This approach allows for improved dose distribution and sparing of adjacent organs at risk (OARs), making it especially suitable for large, deep-seated, or radioresistant tumors [8]. Lattice can be delivered using intensity-modulated radiation therapy (IMRT) or volumetric modulated arc therapy (VMAT) [11]. This approach could potentially enhance the response of bulky tumors through several biological mechanisms, including microvascular tissue alteration, bystander and abscopal effects, and modulation of the immune response [12]. With this technique, it is possible to obtain adequate palliation without altering the patients' quality of life due to a low toxicity profile and rapid onset of symptom control [13]. We report the case of a patient with bulky clear cell renal carcinoma treated with LRT for palliative purposes.

## Case presentation

A 67-year-old male patient was initially evaluated in 2022 at a first medical center for chronic lower back pain radiating to the right flank. Initial imaging studies at that time were within normal limits. In February 2023, he presented with a right supraclavicular mass. Ultrasound revealed an ovoid lymph node measuring 38 × 27 × 32 mm, with an estimated volume of 17.7 cc. An abdominal CT scan showed a cortical mass in the lower pole of the right kidney measuring 83 × 67 × 48 mm (volume: 140 cc), along with a lymph node conglomerate extending from the right diaphragmatic pillar to the iliac bifurcation, measuring 147 × 108 × 190 mm. This mass encompassed the inferior vena cava and left renal vein and displaced the aorta. Two hepatic nodules (11 mm and 13 mm) and a pulmonary nodule in the right anterior segment III (9.3 × 6.3 mm) were also identified. A cervical lymph node biopsy confirmed the diagnosis of clear cell renal carcinoma. The disease was classified as clinical stage IV due to retroperitoneal, pulmonary, hepatic, and supraclavicular metastases. Systemic therapy with sunitinib was initiated and continued for five months. Since the initial evaluation was conducted at another facility, where both imaging and histopathological studies were performed, access to those earlier investigations was limited, and full access to the corresponding reports could not be obtained.

**Figure 1:**
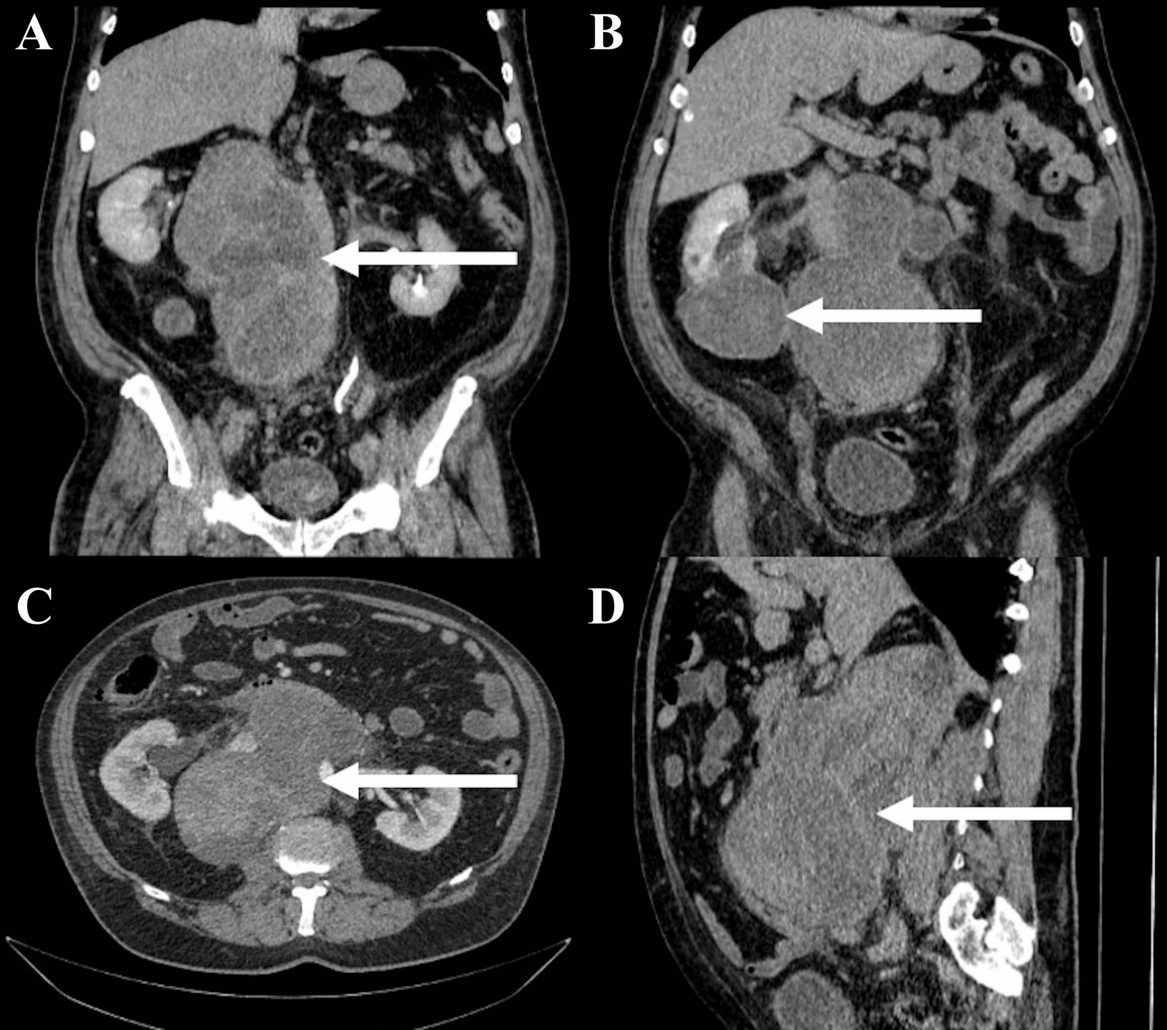
CT of the abdomen and pelvis in coronal (A and B), axial (C), and sagittal (D) planes The images show a large pelvic mass measuring 224x 162 x 111 mm in the longitudinal, transverse, and anteroposterior dimensions, respectively. It completely compresses the inferior vena cava immediately proximal to the inferior vena cava conformation, wraps around the abdominal aorta below the renal arteries, displacing the right renal artery (white arrows A, C, D). There is also a right renal mass in the lower pole measuring 84 x 76 mm (white arrow B) CT: computed tomography

In October 2023, the patient was referred to our institution for presenting symptoms of lower limb and pelvic pain with a pain score of 8/10 despite analgesic treatment, dyspnea, moderate edema in the lower extremities, and difficulty ambulating, arriving in a wheelchair. Initial performance status was assessed with an Eastern Cooperative Oncology Group ECOG score of 2 and a Karnofsky Performance Status (KPS) of 70%. A CT scan revealed a significant increase in the size of the retroperitoneal mass, measuring 224 × 162 × 111 mm in the longitudinal, transverse, and anteroposterior dimensions, respectively. The mass caused complete compression of the proximal inferior vena cava, and enlargement of the right renal lesion was also observed, as shown in Figure 1.

**Figure 2:**
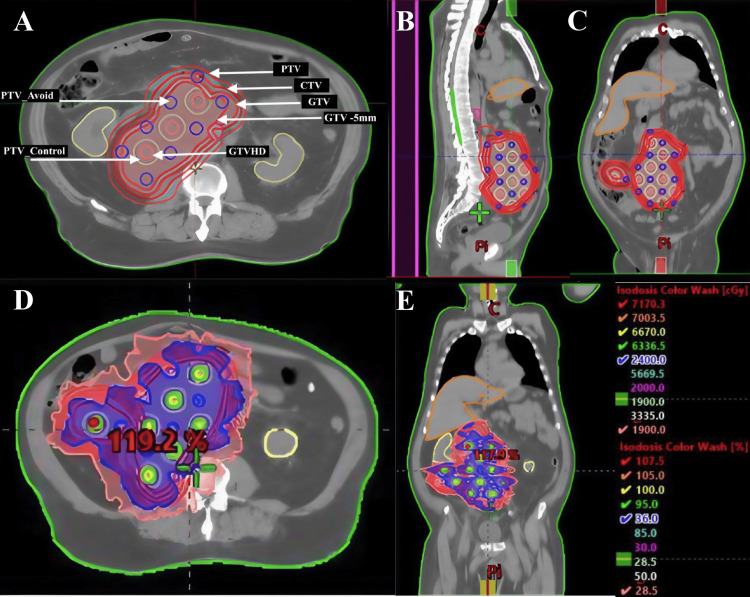
Treatment plan: axial (A), sagittal (B), and coronal (C) planes; dose distribution in axial (D) and coronal (E) planes Images A, B, and C illustrate the outline of the lattice radiotherapy plan, including the geometric arrangement of vertices and tumor volumes. In image A, structures are labeled with white arrows: the high-dose vertices (GTVHD) are shown in pink and are surrounded by PTV_Control, represented as a yellow ring. The avoidance vertices (PTVAvoid) are displayed in blue. The main tumor volumes—GTV, CTV, and PTV—as well as the inner optimization shell (GTV -5mm), are contoured in red. Images D and E display the dose distribution after VMAT planning, with pink representing 19 Gy and yellow representing 66.7 Gy CTV: clinical target volume; GTV: gross target volume; PTV: planning target volume; VMAT: volumetric modulated arc therapy

Due to the pain symptoms and obstructive manifestations caused by the mass effect on the inferior vena cava, as well as the high risk of pulmonary thromboembolism (PTE), the patient was referred for urgent decompressive radiotherapy. LRT was initiated, delivering 66.7 Gy in five fractions to the high-dose regions (30 apexes), and 20 Gy in the same number of fractions to the valley zones, corresponding to the peripheral areas adjacent to OARs. All target volumes were contoured following the departmental protocol, which incorporates the dose planning principles described by Duriseti et al. [8]. Briefly, spherical high-dose vertices of 1.5 cm in diameter were arranged throughout the gross tumor volume (GTV) and designated as GTVHD. These were arranged geometrically with 6 cm center-to-center spacing and repeated every 3 cm in the superior-inferior direction. These vertices were alternated with avoidance vertices (PTVAvoid) of equal size, maintaining a minimum 3 cm distance between high-dose and avoidance structures. No high-dose vertices were placed within 1.5 cm of any OAR or outside a 5 mm contraction of the GTV (defined as GTV-5mm) to minimize unintended dose to healthy tissues. Additionally, a PTV_Control was created by expanding GTVHD by 8 mm, and was used to assess dose fall-off between adjacent GTVHD vertices as illustrated in Figure 2.

**Figure 3:**
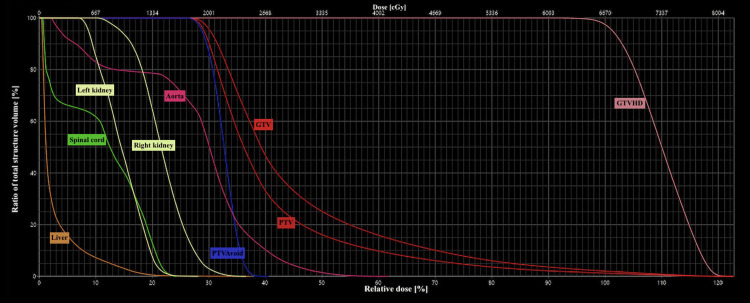
DVH depicting target volumes and organs at risk in the lattice radiotherapy plan The figure displays the DVH curves for GTV, high-dose apexes (GTVHD), PTV, the PTVAvoid structure (valley zone), and the organs at risk. The structures are color-coded as indicated in the figure DVH: dose-volume histogram; GTV: gross target volume; PTV: planning target volume

Clinical target objectives and constraints are summarized in Table 1, and the dose-volume histogram (DVH) is shown in Figure 3.

**Table 1:** Target dose and organs at risk dose constraints achieved in lattice treatment GTV: gross target volume; PTV: planning target volume

Plan			Lattice		
Total dose			6670.0 cGy		
Structure name	Clinical Goal summary		1	0	18
GTV	P1	D 0.0 cm3 ≤8000 cGy	8184.36 cGy		
GTVHD (apex zones)	P1	V 6330 cGy ≥95.0%	99.87 %		
PTV	P1	V 1900 cGy ≥95.0%	97.42 %		
PTVAvoid (valley zones)	P1	Dmedia ≥2000 cGy	2175.44 cGy		
	P1	Dmedia ≤2400 cGy	2175.44 cGy		
	P1	D 100.0 cm3 ≤1900 cGy	1674.73 cGy		
Aorta	P2	D 10.0 cm3 ≤4700 cGy	2467.08 cGy		
	P2	D 0.0 cm3 ≤5300 cGy	4116.79 cGy		
Liver	P2	V 3000 cGy ≤70.0%	0.00 %		
	P2	V 2100 cGy ≤700.0 cm3	0.58 cm3		
Spinal cord	P2	D 1.2 cm3 ≤1450 cGy	1424.59 cGy		
	P2	D 0.4 cm3 ≤2300 cGy	1492.73 cGy		
	P2	D 0.0 cm3 ≤3000 cGy	1620.37 cGy		
Right kidney	P2	D 66.0% ≤2300 cGy	1322.83 cGy		
	P2	D 35.0% ≤1800 cGy	1550.31 cGy		
	P2	V 2300 cGy <66.0%	0.12 %		
Left kidney	P2	D 66.0% ≤2300 cGy	845.10 cGy		
	P2	D 35.0% ≤1800 cGy	1089.77 cGy		
	P2	V 2300 cGy <66.0%	0.00 %		

**Figure 4:**
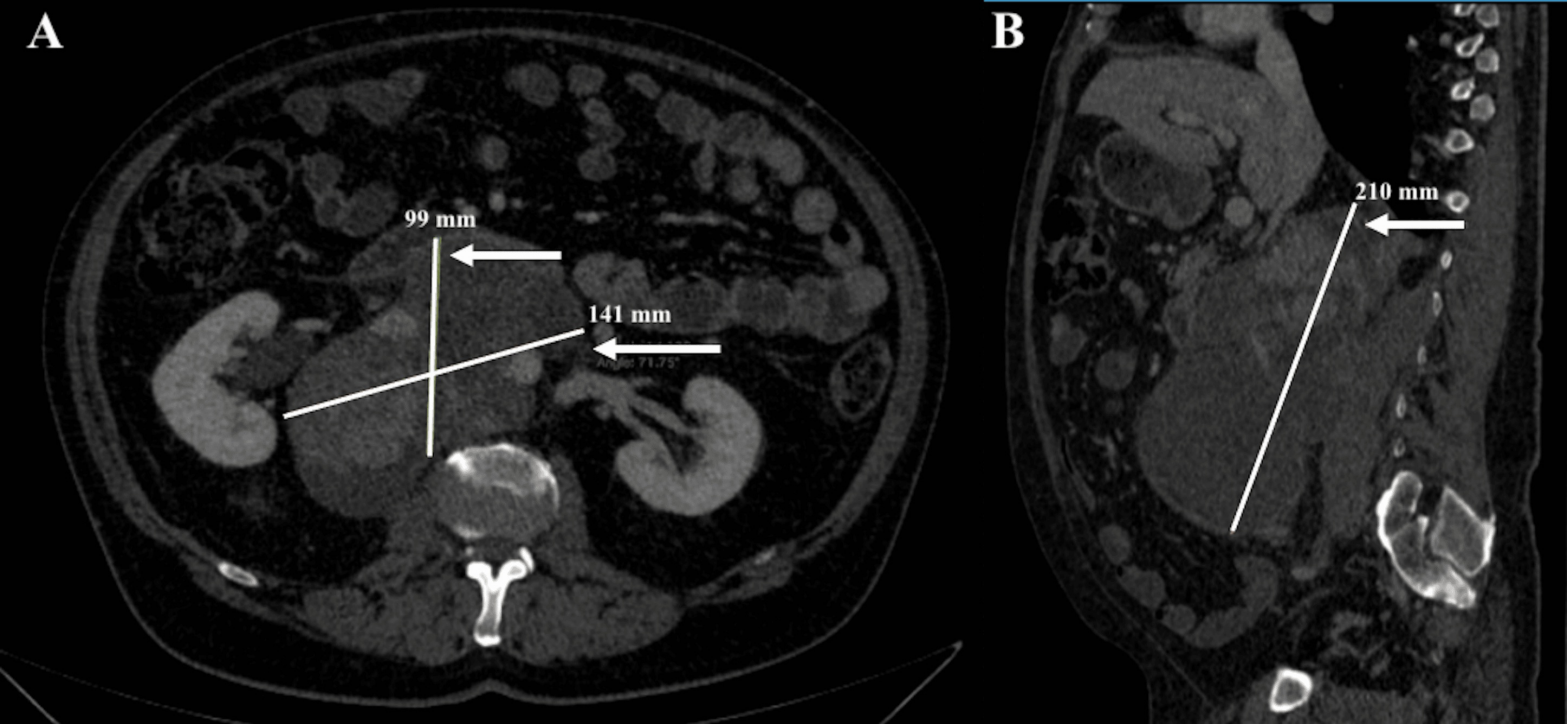
Abdominal and pelvic CT scan at the first follow-up: axial (A) and sagittal (B) planes The image shows a 6.2% reduction in the volume of the retroperitoneal lesion, currently measuring 210 × 141 × 99 mm in the longitudinal (B), transverse, and anteroposterior axes (A), respectively (delineated within the white lines), compared to the previous measurement of 224 × 162 × 111 mm CT: computed tomography

At the end of the radiotherapy treatment, the patient showed an immediate symptomatic response with no evidence of acute toxicity. Notably, no anti-nausea medication was required before or during the entire course of treatment. Both acute and late toxicities were assessed and graded according to the Common Terminology Criteria for Adverse Events (CTCAE) Version 5.0, with no acute gastrointestinal or general symptoms observed. Clinically, there was significant improvement, including substantial relief from obstructive and pain-related symptoms. The pain score decreased from 8/10 to 2/10, accompanied by a marked reduction in pelvic and lower limb discomfort, complete resolution of lower extremity edema, and restored mobility, enabling ambulation without assistance. During radiotherapy, the patient did not receive systemic therapy; however, once radiotherapy concluded, systemic treatment was resumed with a change in regimen due to therapeutic failure, switching from sunitinib to axitinib plus pembrolizumab as second-line therapy. The tomographic control at two months showed a decrease in the retroperitoneal lesion of up to 6.2% compared to the previous study. This decrease in the size of the abdominal mass is shown in Figure 4.

**Figure 5:**
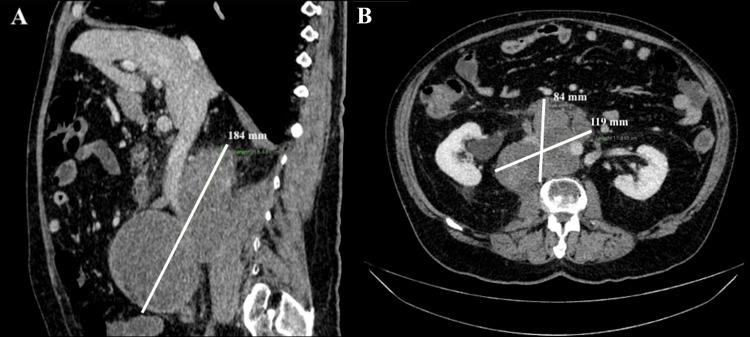
Abdominal and pelvic CT at eight months in sagittal (A) and axial (B) planes The images show a greater decrease in the size of the retroperitoneal lesion eight months after the application of the Lattice radiotherapy technique, with current dimensions 184 × 119 × 82 mm in the longitudinal (A), transverse, and anteroposterior axes (B and C), respectively (delineated within the white lines) CT: computed tomography

Finally, a CT scan of the abdomen and pelvis performed eight months after radiotherapy showed that the retroperitoneal mass continues to decrease in size up to 17.8% when compared to the initial scans (Figure 5).

On clinical assessment as of December 30, 2024, the patient remains in good functional status, with an ECOG performance score of 1 and a KPS of 90%. No chronic toxicities were observed. The patient has had 12 months of progression-free survival (PFS) and complete resolution of pain (pain score 0/10), without the need for any pain medication, while continuing systemic therapy.

## Discussion

While radiotherapy has historically been used for palliative purposes, its practice in renal cell carcinoma has been limited by radioresistance [14]. However, emerging evidence suggests that radiotherapy, particularly stereotactic ablative body radiation (SABR), may offer a promising therapeutic option for patients with early-stage inoperable or metastatic disease [1]. Since SABR is generally restricted to lesions smaller than 5 cm, alternative radiotherapy techniques are essential for the effective management of larger tumor masses while maintaining a low toxicity profile [15]. In this context, SFRT has emerged as a potentially valuable strategy for treating bulky tumors [15]. This technique was introduced in 2010 and then studied in 2020 by Wu et al., who proposed that 2D GRID radiotherapy could be extended to a 3D configuration [16]. 2D GRID radiotherapy is a technique that delivers a highly nonuniform dose distribution, consisting of alternating regions of high and low dose. It is primarily used for large, unresectable tumors that are difficult to manage with conventional radiation therapy techniques [17]. The Lattice technique, a specific form of SFRT, creates a spatial dose distribution consisting of high-dose vertices and low-dose valleys, allowing the delivery of ablative doses to selected tumor regions while minimizing exposure to surrounding healthy tissues and OARs [12].

Over recent years, LRT has gained increasing attention, with a growing number of case reports demonstrating its clinical utility [18]. In 2015, Blanco Suarez et al. reported improved outcomes in a patient with ovarian carcinosarcoma treated with LRT combined with standard chemotherapy and fractionated radiotherapy [19]. Favorable responses have also been described in various tumor types, including non-small cell lung cancer (NSCLC) [20], bulky metastases of cutaneous squamous cell carcinoma [21], prostate cancer [22], and gynecologic malignancies [23]. A systematic review of this technique by Lori et al. identified a total of 81 patients with 84 lesions ranging from 63.2 cc to 3713.5 cc. The results indicated that LRT is both safe and effective. Among patients who did not achieve a complete response at three to six months, a mean tumor volume reduction of approximately ≥50% was observed when used with palliative and cytoreductive intent [15]. Although this review did not include patients with RCC, it supports the utility of LRT in managing bulky tumors with limited toxicity.

Our patient received LRT in five fractions with a total of 66.7 GY. The initial size of the abdominal metastatic lesion (224 × 162 × 111 mm) was reduced by 6.2% at two months and by 17.8% at eight months, with concurrent systemic therapy using axitinib and pembrolizumab. While the tumor reduction did not exceed 50%, the primary goal was palliative, and the clinical benefit was evident: the patient experienced relief of abdominal pain, an ECOG performance status of 1, and a Karnofsky score of 90%, indicating a significant improvement in quality of life. An additional point of interest is the potential synergy between LRT and systemic therapies, including chemotherapy, targeted agents, and immunotherapy. Many systemic drugs function as radiosensitizers, and their concurrent use with LRT may enhance therapeutic response. Furthermore, the potential for the abscopal effect, an immune-mediated response triggered by localized ablative radiation, could further improve outcomes when combined with immunotherapy [12,15].

Despite the growing number of reports and case series, level I evidence supporting the use of LRT remains scarce. Current clinical guidelines do not include specific recommendations for this technique, and the broader literature remains limited [24]. Most available studies comprise individual case reports or small series, and key questions regarding optimal dose distribution, fractionation schemes, and geometric configurations remain unanswered. These aspects are currently being investigated in preclinical and early-phase clinical studies [18]. A recent study, LATTICE_01, evaluated the efficacy and safety of metabolic-guided LRT in 30 patients with stage IV disease and 31 bulky lesions. The study reported an overall clinical response rate of 89%, a one-year overall survival rate of 86.4%, and grade ≥1 acute toxicities in 20% of patients [13]. Similarly, the phase I LITE SABR M1 trial assessed acute toxicity and quality of life in 20 patients (22 tumors) with lesions larger than 5 cm. No grade 3 toxicities were observed within 90 days, and only one case of grade 4 toxicity was reported [25]. However, to date, no randomized controlled trials or comparative observational studies have evaluated the benefits of LRT compared to conventional palliative radiotherapy.

Evidence supporting the use of this technique in clear cell renal carcinoma remains scarce. Nonetheless, the clinical response observed in this case, marked symptom improvement without associated toxicity, offers valuable insights into the potential role of LRT in this context. The clinical benefit was evidenced by improvement in functional status, the patient's recovery to perform routine daily activities, and a marked reduction in pain as previously described. Among the expected complications related to LRT, tumor lysis syndrome has been described [15]; however, no clinical or laboratory evidence of this complication was observed in the present case. This report contributes to the expanding body of literature on the application of this novel technique in oncologic scenarios where current clinical evidence is limited.

## Conclusions

This clinical case highlights the therapeutic potential of LRT in the palliative management of large metastatic renal tumors, particularly in patients with advanced clear cell renal carcinoma. Despite the known radioresistance of this tumor type, the delivery of ablative doses through SFRT enabled progressive tumor volume reduction without associated toxicity, while significantly improving the patient’s symptoms and quality of life. The combination with systemic therapies such as axitinib and pembrolizumab might have contributed to the observed effects, indicating a potential but not yet established synergistic interaction between LRT and immunotherapy. Although the results are encouraging, this report also underscores the need for controlled clinical trials to validate the efficacy, safety, and optimal protocols for this technique in renal cancer. We believe this report adds to the growing body of evidence supporting advanced radiotherapy approaches in complex oncologic scenarios, offering new perspectives on the treatment of bulky tumors.
